# *In Silico* Identification of Mimicking Molecules as Defense Inducers Triggering Jasmonic Acid Mediated Immunity against *Alternaria* Blight Disease in *Brassica* Species

**DOI:** 10.3389/fpls.2017.00609

**Published:** 2017-04-25

**Authors:** Rajesh K. Pathak, Mamta Baunthiyal, Rohit Shukla, Dinesh Pandey, Gohar Taj, Anil Kumar

**Affiliations:** ^1^Department of Molecular Biology and Genetic Engineering, College of Basic Sciences and Humanities, G. B. Pant University of Agriculture and TechnologyPantnagar, India; ^2^Department of Biotechnology, G. B. Pant Engineering CollegePauri Garhwal, India; ^3^Molecular and Structural Biophysics Laboratory, Department of Biochemistry, North Eastern Hill UniversityShillong, India

**Keywords:** *Brassica*, *Alternaria* blight, COI1, jasmonic acid, virtual screening, molecular dynamics simulation

## Abstract

*Alternaria brassicae* and *Alternaria brassicicola* are two major phytopathogenic fungi which cause *Alternaria* blight, a recalcitrant disease on *Brassica* crops throughout the world, which is highly destructive and responsible for significant yield losses. Since no resistant source is available against *Alternaria* blight, therefore, efforts have been made in the present study to identify defense inducer molecules which can induce jasmonic acid (JA) mediated defense against the disease. It is believed that JA triggered defense response will prevent necrotrophic mode of colonization of *Alternaria brassicae* fungus. The JA receptor, COI1 is one of the potential targets for triggering JA mediated immunity through interaction with JA signal. In the present study, few mimicking compounds more efficient than naturally occurring JA in terms of interaction with COI1 were identified through virtual screening and molecular dynamics simulation studies. A high quality structural model of COI1 was developed using the protein sequence of *Brassica rapa*. This was followed by virtual screening of 767 analogs of JA from ZINC database for interaction with COI1. Two analogs viz. ZINC27640214 and ZINC43772052 showed more binding affinity with COI1 as compared to naturally occurring JA. Molecular dynamics simulation of COI1 and COI1-JA complex, as well as best screened interacting structural analogs of JA with COI1 was done for 50 ns to validate the stability of system. It was found that ZINC27640214 possesses efficient, stable, and good cell permeability properties. Based on the obtained results and its physicochemical properties, it is capable of mimicking JA signaling and may be used as defense inducers for triggering JA mediated resistance against *Alternaria* blight, only after further validation through field trials.

## Introduction

*Brassica* species are one of the second largest oilseed producing crops in the world after soybean [*Glycine max* (L.) Merr.], surpassing sunflower (*Helianthus annuus* L.), peanut (*Arachis hypogaea* L.), and cottonseed (*Gossypium hirsutum* L.) during the last several decades (FAO, 2010; Agricultural Outlook, 2010–2019). Diseases and insect pest are important limiting factors, which restrict the expansion of cultivation and abate the productivity of *Brassica* crops. More than 30 diseases are known to occur on *Brassica* crops in India (Saharan, 1992), majority of which are caused by different fungal pathogens, whereas viral and bacterial diseases have minute effect on their yield. *Alternaria* blight caused by *Alternaria brassicae* and *Alternaria brassicicola* is the most common and destructive disease of *Brassica* throughout the world which can result in yield reductions of up to 36% (Duczek et al., 1998). In India, 10–70% of yield losses were reported in different region across the country (Kolte et al., 1987; Ram and Chauhan, 1998). Many efforts have been made by scientists to develop disease resistant transgenic plants with limited success to develop complete *de novo* resistance against *Alternaria* blight due to lack of knowledge about the resistance gene homologs (Mondal et al., 2003; Taj et al., 2004; Marmath et al., 2011; Kumar et al., 2014). It has been realized that mere use of transgenic technology is no longer useful for development of resistance toward diseases like *Alternaria* blight (Kumar et al., 2015). So, there is required use of innovative approaches in agricultural sciences to tackle such type of problems.

There are various choices available for the farmers to protect their *Brassica* crops from *Alternaria* blight disease, which includes cultivation of resistant variety, biological control, crop rotation, and use of fungicides. However, these methods have certain disadvantages. For example, the use of fungicides to manage the disease is both biohazardous and ecounfriendly. The pathogen constantly changes its nature therefore; the resistant cultivars may become susceptible with the time (Chaudhary et al., 2001). Recently, a new technology for management of crop plants disease is being adopted where host plants develop own defense system which are activated with the aid of low molecular weight natural or synthetic molecules (Cohen et al., 1999; Pathak et al., 2016). Such molecules also known as defense inducers could serve as promising alternatives to convential biohazardous pesticides in managing the diseases. It has already been reported that the exogenous use of low molecular weight molecules including phytohormones such as jasmonic acid (JA), salicylic acid and its functional analoges as well as phytoalexins have been shown to trigger systemic acquired resistance in plant systems against a wide range of plant–pathogen interactions (Thaler et al., 2004; Pedras et al., 2009; Mandavia et al., 2012; Kazan and Lyons, 2014; Bektas and Eulgem, 2015; Pathak et al., 2016). Besides being involved in developmental responses, JA is reported to play significant role in providing defense responses to crop plant during many plant–pathogen interactions (Yan et al., 2009; Pandey et al., 2016). A great deal of knowledge about the action of JA emerged from various experimentation and data analysis of *Arabidopsis* mutants with alterations in the biosynthesis of JA as well as signal transduction. Coronatine insensitive1-1 (coi1-1) is one such mutant, which exhibits the male sterility, defects in the expression of JA-regulated genes and resistance to JA inhibition of root growth (Feys et al., 1994). The COI1 is an F-box protein which is associated with *Arabidopsis* Skp1-like 1, *Arabidopsis* Skp1-like 2 (Xie et al., 1998; Liu et al., 2004), cullin 1, as well as ring-box protein 1 to make an E3 ubiquitin ligase called as the SCF^COI1^ complex (Xu et al., 2002; Ren et al., 2005). It is well-defined in plants systems that E3 ubiquitin ligases are engaged in the ubiquitination of target proteins for subsequent degradation *via* the 26S proteasome (Moon et al., 2004). COI1 has been recently found to act as JA receptor (Raya-González et al., 2012) which is capable to interact with some proteins, including jasmonate ZIM-domain (JAZs) protein (Chini et al., 2007; Thines et al., 2007; Yan et al., 2007), ASK1, ASK2, Rbx1, and Cullin1 (Xu et al., 2002; Wang et al., 2005), which were found to be a part of the complex that binds with JAs. The JA binding assays in previously conducted studies were carried out with crude plant extracts or partially purified proteins (Thines et al., 2007; Katsir et al., 2008; Melotto et al., 2008; Fonseca et al., 2009), Recent studies on *Arabidopsis thaliana* have demonstrated that the COI1 (coronatine insensitive 1) directly interacts to JA-Ile/Coronatine (COR) and works as a receptor for JA (Yan et al., 2009) and plays vital role in defense responses through signal transduction. There is a growing demand to identify a molecule which is more efficient than JA, in terms of it’s affinity with JA receptor and such mimicking molecules could produce same level of defense response during plant–pathogen interaction.

COI1 has the structural traits for binding to jasmonoyl-isoleucine (JA-Ile)/coronatine (COR), which was identified through immobilized JA approach, surface plasmon resonance technology and photoaffinity labeling technology (Yan et al., 2009). It is determined as an intracellular receptor for JA signal with a hormone binding site (Sheard et al., 2010), which may serve as a potential molecular target for identification of mimicking molecules.

In view of the above facts there is a need of computational approaches to decipher the role of mimicking molecules of JA signaling as defense inducer which could serve as viable alternative of fungicides for the management of recalcitrant disease like *Alternaria* blight in *Brassica* in ecofriendly manner. The present study is centered on the discovery of novel agriculturally important molecules through docking, virtual screening and molecular dynamics simulation studies. The newer approach of induction of *de novo* resistance through such identified molecules will enable better crop protection of *Brassica* spp. and help in fulfilling the growing demands of oilseeds.

## Materials and Methods

### Sequence Retrieval and Analysis

The nucleotide (Accession no., GU263836.1) and amino acid (Accession no., ADK47027.1) sequences of the *Brassica rapa* COI1 were retrieved from National Centre for Biotechnology Information^1^ database. MATLAB Bioinformatics toolbox^2^ was used to analyze the sequences to determine the basecount, dimercount, nucleotide density, AT-GC density, and protein composition.

### Primary Structure Analysis

The primary structure of protein was studied using ProtParam tool^3^ (Gasteiger et al., 2005) of Expasy Server. Various physico-chemical parameters such as molecular weight, isoelectric point, instability index, aliphatic index, and grand average hydropathy (GRAVY) were computed. The secondary structure prediction of the COI1 was done using SOPMA tool^4^. It is a self-optimized prediction method which has been explained to improve the success rate in secondary structure prediction of the given protein sequence (Geourjon and Deléage, 1995).

### Homology Modeling of COI1

The 596 amino acid residues in length, COI1 protein of *B. rapa* was subjected to BLASTp analysis against RCSB PDB^5^ to identify suitable template for comparative protein structure modeling and furthermore functional prediction. The results of BLASTp suggested that 3OGK (B-chain) is the most appropriate template for COI1 protein model building, having the highest sequence identity, query coverage, and less E-value (Altschul et al., 1990). As comparative modeling relies on a sequence alignment between target sequence and the template sequence whose structure has been experimentally determined, the 3D structure of target protein using its template was generated by Modeller 9.13 tool; based on template-target alignment, five different 3D models of COI1 were generated by Modeller9.13. These theoretical structural models of COI1 were ranked based on their normalized discrete optimized protein energy (DOPE) scores. The model with the lowest DOPE score was considered as the best model (Webb and Sali, 2014).

### Evaluation of Structural Model

The quality of rough COI1 model was evaluated by a number of tools to test the stability and reliability of model. PROCHECK (Laskowski et al., 1993) investigation, which quantifies the residues in available zones of Ramachandran plot, was used to assess the stereo chemical quality of the model. ERRAT (Colovos and Yeates, 1993) tool, which finds the overall quality factor of the protein, was used to check the statistics of non-bonded interactions between different atom types. Similarly, to determine the compatibility of the atomic model (3D) with its own amino acid sequence, the VERIFY_3D (Luthy et al., 1992) program was used. All the above analysis was carried out using Structural Analysis and Verification Server ^6^ which having assembled program. Swiss PDB Viewer 4.1.0^7^ was used for the energy minimization of the predicted COI1 protein. The COI1 model was further subjected to Structural Analysis and Verification Server to evaluate its quality, after energy minimization and before energy minimization. ProSA (Wiederstein and Sippl, 2007) tool was employed in the refinement and validation of the minimized structure to check the native protein folding energy of the model by comparing the energy of the model with the potential mean force derived from a large set of known protein structure. The super imposition of the proposed model of COI1 protein with its closest-structural homolog was carried out using Chimera 1.11 (Pettersen et al., 2004).

### Binding Site Prediction and Analysis

Usually, binding site is highly conserved among closely related proteins. The functional activity of a protein is performed by a highly conserved group of amino acid residues within the binding site. Active site identification included the superimposition of the model with template that provided integrity of the homology model and assisted in positioning conserved active site residues. The binding site residues of COI1 was predicted by COACH server^8^, It is based on meta-server approach to investigating the ligand binding site in target protein structure using comparison with complementary binding-specific substructure and sequence profile alignment (Yang et al., 2013).

### Ligand Preparation and Virtual Screening

Literature studies were followed to retrieve the 3D structure of JA (CID: 5281166) from pubchem database^9^ and subsequently utilized the smile notation of JA to search the ZINC database^10^ with cut off of 50% structural identity (Irwin and Shoichet, 2005). We have found 767 JA analogs and downloaded these compounds in sdf file format. Further local python script were used to convert sdf into pdb and subsequently there was prepared pdbqt file of each ligand for virtual screening. Virtual screening was performed against COI1 using AutoDock vina (Trott and Olson, 2010).

### Molecular Dynamics Simulation

To investigate the stability and dynamics of the predicted protein model and docked complexes, MD simulations were carried out using GROMOS96 53A6 force field (Walter et al., 1999) in GROMACS 5.0.1 (Groningen Machine for Chemical Simulations) suite (Van Der Spoel et al., 2005). All the systems were solvated in a cubic box by using SPC water model. System showed -3 negative charges so for neutralizing the systems, 3 Na^+^ ions were added. The COI1 and COI1-JA, COI1-ZINC43772052 as well as COI1-ZINC27640214 complexes were simulated at 50 ns time scale. In the first system COI1 was simulated for analyzed its dynamic behavior and stability without ligand. After that the ligand parameters of JA, ZINC43772052 and ZINC27640214 were generated by using ProDRG server (SchuÈttelkopf and Van Aalten, 2004), and employed for MDS studies with COI1 for predicting its perturbation with small ligand molecule and analyzed its stability in complex form to understands the mimicking nature of JA and its structural analogs. Steepest energy minimization was performed for all the systems to give the maximum force below 1000 kJ/mol/nm for removing the steric clashes. Ewald summation method with PME implementation were used for calculating the electrostatic forces (Darden et al., 1993). Columb interaction and Lennard-Jones were calculated within a cut-off radius of 1.0 nm (Hess et al., 1997). For predicting the short-range non-bonded interaction, 10 Å cut-off distance was used. 1.6 Å Fourier grid spacing was used for the PME method for long-range electrostatics. After energy minimization the systems were employed for 1 ns position restraint simulation under NVT and NPT condition for relaxation of the solvent molecules and finally 50 ns MDS study were carried out for all above four systems. The RMSD, RMSF, Hydrogen bonds and principal component analysis were calculated by g_rms, g_rmsf, g_hbond, g_covar tools within the gromacs utilities (Van Der Spoel et al., 2005).

### Free Energy Calculation

Binding free energy of protein–ligand complex was calculated by using g_mmpbsa tool (Kumari et al., 2014). Molecular mechanics Poisson–Boltzmann surface area (MMPBSA) approach was used by this software to calculate the binding free energy of complex. Molecular mechanics potential energy (electrostatic + Van der Waals interaction) and the free energy of solvation (polar + non-polar solvation energies) was calculated by this tool but this is not able to calculate the entropy of the system. Last 10 ns trajectory was considered for free energy calculation.

## Results

### Sequence Analysis

**FIGURE 1:**
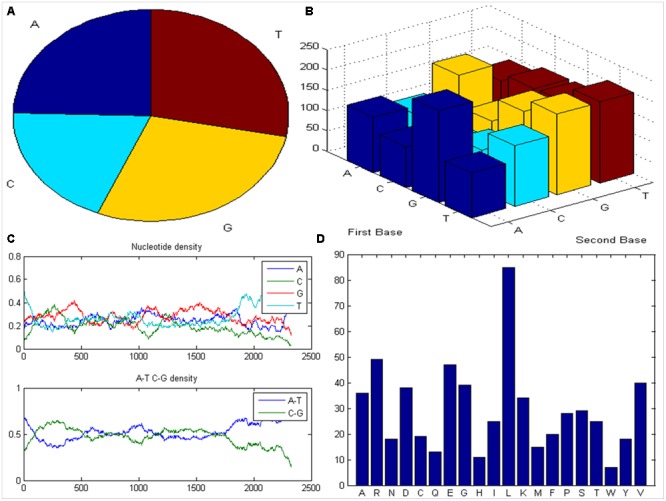
**Analysis of COI1 sequence. (A)** Distribution of nucleotide **(B)** bar chart of dimmers **(C)** nucleotide density and A-T-C-G rich region **(D)** content of amino acid present in COI1.

The publically accessible complete nucleotide sequence of the COI1 (2327 bp) from *B. rapa* L., which is expected to play vital role in defense responses during pathogenesis of *Alternaria* blight, was downloaded from NCBI and subjected to MATLAB Bioinformatics toolbox^2^ to visualize the distribution of nucleotide using pie chart (**Figure 1A**) and dimmers were displayed in bar chart (**Figure 1B**). Subsequently nucleotide density and A-T-C-G density were plotted to determine the location of A-T and C-G rich region present in the sequence (**Figure 1C**). After determining the content of amino acid present in the protein sequence of COI1, we have found the high content of Leucine, Arginine, Glutamic acid and Valine, and this information was plotted using bar graph (**Figure 1D**). The ProtParam tool was used to compute a molecular weight of 67779.62 for COI1. The isoelectric point (pI) is the pH at which the surface of protein is covered with charge but net charge of protein is zero. At pI, proteins are stable and compact. The COI1 had a pI of 6.63, indicating its acidic nature (pI < 7.0). The aliphatic index (AI) is called as the relative volume of a protein occupied by aliphatic side chains such as alanine, valine, leucine, and isoleucine. It is considered as a positive factor for enhancement of the thermal stability of globular proteins (Ikai, 1980). Since the aliphatic index of COI1 was very high (i.e., 97.48), it indicates that COI1 may be stable for a wide range of temperatures. The instability index gives an estimate regarding the stability of protein in a test tube. A protein having instability index less than 40 is predicted as stable, whereas, a value above 40 predicts that the protein may be unstable (Guruprasad et al., 1990). The instability index of COI1 was calculated as 42.18, which indicated it’s unstable nature. The Grand average of hydropathicity (GRAVY) of COI1 was very low (-0.209), which indicate its high affinity for water.

### Homology Modeling of COI1

**FIGURE 2:**
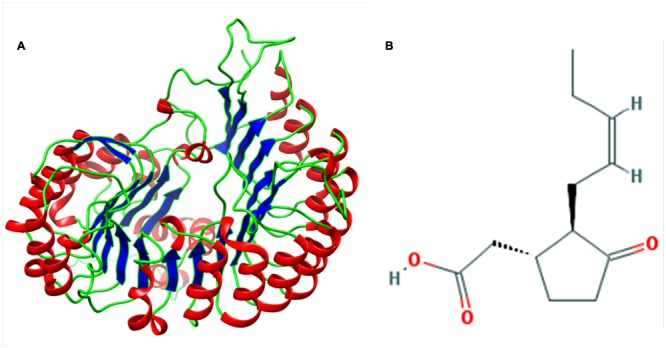
**(A)** Predicted three-dimensional structure of COI1. **(B)** Structure of jasmonic acid (JA).

The result of BLASTp search revealed three putative templates (PDB id: 3OGK, 2P1M, and 3O61) of high-level identity with the target sequence, as shown in **Table 1**. These templates are the crystal structures of COI1-ASK1 in complex with coronatine and an incomplete JAZ1 degron, and TIR1-ASK1 complex structure from *Arabidopsis thaliana*, and GDP-mannose hydrolase complex from *Escherichia coli* (Tan et al., 2007; Sheard et al., 2010; Boto et al., 2011). Based on the BLAST results PDB ID: 3OGK with a resolution of 2.8 A°, is the best template for comparative modeling. Modeller 9.13 generated five rough models of COI1. Out of these five different models, the model with the lowest DOPE score was considered to be thermodynamically stable and selected for further refinement and validation (Eswar et al., 2008) (**Figure 2A**). To judge the conservedness among the secondary structure components, the secondary structure of the COI1 and the template was predicted and compared from their primary sequence. The secondary structure comparison between the target and template showed strong homology across the entire length, as shown in **Table 2**. The conservation of the secondary structure disclosed the reliability of our proposed model predicted by Modeller tool based on the target-template alignment.

**Table 1:** Templates selected for comparative model building of COI1 through BLAST search against RCSB PDB.

Templates (PDB id with their chain)	Total score	Query coverage (%)	E-value	% of identity	Resolution (A^◦^)
3OGK_B	965	99	0.0	81	2.8
2P1M_B	247	97	1e-73	31	1.8
3O61_A	32.0	9	1.2	35	2.45

**Table 2:** Secondary structure comparison of COI1 and its template with respect to percentage of amino acid residues.

Target/ template	Helix	Strand	Coil	Total number of amino acids
COI1 (target)	280 (46.98%)	101 (16.95%)	215 (36.07)	596
3OGK_B	300 (50.68%)	77 (13.01%)	215 (36.32)	592

### Model Assessment and Validation

**FIGURE 3:**
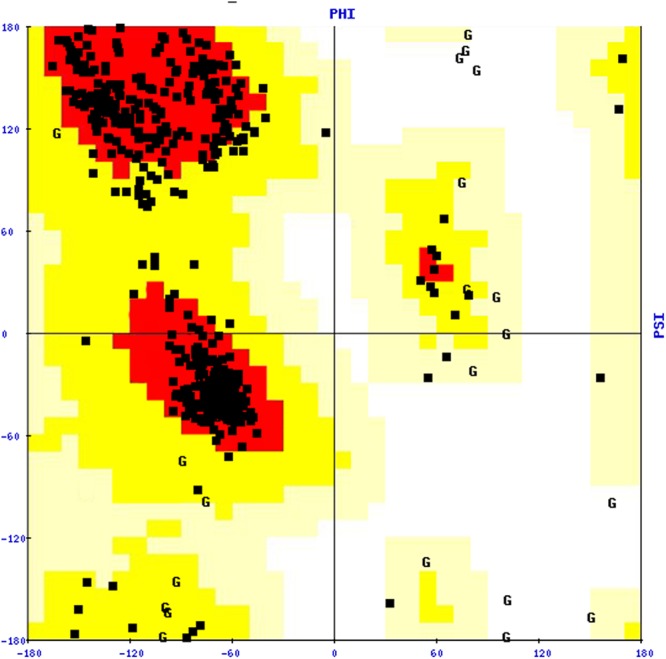
**Ramachandran plot of the COI1 model.** The plot was constructed with the PROCHECK tool.

To stabilize the stereochemical properties of the COI1 model, energy minimization was done using the steepest descent algorithm to remove the bad contacts between protein atoms. The energy minimization was conducted in vacuo with the GROMOS96 43B1 parameters through SPDB Viewer 4.1.0 (Guex and Peitsch, 1997). Further the stability of the model was validated through the Structural Analysis and Verification Server^11^, the reliability of the backbone of torsion angles φ and Ψ of the model was evaluated by PROCHECK, which computes the amino acid residues fall in the existing zones of Ramachandran plot, as shown in **Figure 3**. The Ramachandran plot analysis for the COI1 model showed that 88.0% residues fell in the most favored regions, 10.9% residues were in additional allowed regions, 1.1% residues were in the generously allowed regions and no residue was in disallowed regions (**Table 3**).

**Table 3:** Ramachandran plot statistics of COI1 model.

Ramachandran plot statistics	Percentage of amino acid residues
Residues in most favored regions	88.0
Residues in additionally allowed regions	10.9
Residues in generously allowed regions	1.1
Residues in disallowed regions	0.0

**FIGURE 4:**
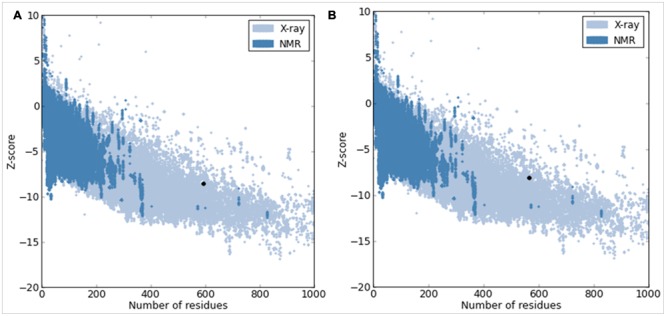
**Protein Structure Analysis (ProSA) of model COI1. (A)** Overall quality of COI1 model showing a z-score of –8.59 (Native conformation to its template). **(B)** Overall quality of template (3OGK_B) model showing a z-score of –8.13.

The quality of our predicted model COI1 was further supported by a high ERRAT score of 81.122 (a value of ∼95% shows high resolution), which signified acceptable protein environment (Colovos and Yeates, 1993). The VERIFY-3D results of the COI1 model showed 93.62% of the residues had an averaged 3D-1D score > = 0.2, indicating the reliability of the model. The PROVE program was used to compute the average magnitude of the volume irregularities with respect to the Z-score root mean square deviation (RMSD) of the COI1 model. The Z-score Root Mean Square values of the model and template were 1.626 and 1.866, respectively (a Z-score RMS value of ∼1.0 showed good resolution of structures). WHAT IF tool examined the coarse packing quality, anomalous bond length, packing quality, planarity, collision with symmetry axis and the distribution of omega angles, proline puckering as well as anomalous bond angles of the model protein structure, reflecting its acceptance of good quality. Further the reliability of COI1 model was confirmed by ProSA. The energy profile of the model and the Z-score value were obtained, which calculates the interaction energy per residue by using a distance-based pair potential. The ProSA analysis of the model COI1 (**Figure 4A**) achieved a Z score of -8.59 and that of template was -8.13 (**Figure 4B**) (where the energy of ProSA in negative reflects the reliability of the model) showed good quality of the model. The quality of the model was also evaluated by comparing the predicted structure with experimentally determined structure by superimposition and atoms RMSD assessment using Chimera 1.11 (Pettersen et al., 2004), which indicates that the predicted model is reasonably good and quite similar with template.

### Binding Site Analysis and Generation of Grid Box

Investigating the binding sites found in predicted protein structure is a challenging task; many efforts have been made to develop some tools that can successfully explore the cavities for binding affinity prediction and scoring with ligand(s) through molecular docking (Wei et al., 2002). COACH for protein–ligand binding site prediction was used to identify consensus binding site area (Yang et al., 2013). The size of grid box was generated as 26, 32, 26, for X, Y, and Z axis on the basis of predicted binding site area as well as the energy range was kept as 4 which is default setting of AutoDock tool (Morris et al., 2008). This grid box size of COI1 was used to performed interaction studies with JA and its analogs through virtual screening.

### Virtual Screening and Analysis of Protein–Ligand Complex

**FIGURE 5:**
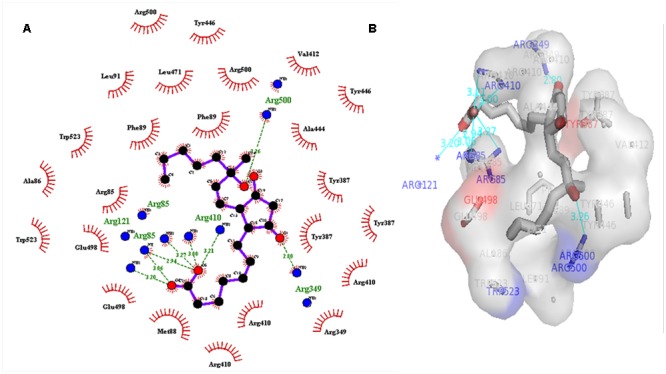
**(A)** 2D, and 3D **(B)** representation of docked structure of COI1 generated by Ligplot and PyMol tool; The 2D representation of 3D structure depicted H-bond interaction of ZINC27640214 with COI1 amino acid residues, ARG85, ARG121, ARG349, ARG410, and ARG500 (green line) whereas the amino acid residues such as ARG85, ALA86, MET88, PHE89, LEU91, ARG349, TYR387, ARG410, VAL412, ALA444, TYR446, LEU471, GLU498, ARG500, and TRP523 were interacted through hydrophobic bonding (in red color).

**FIGURE 6:**
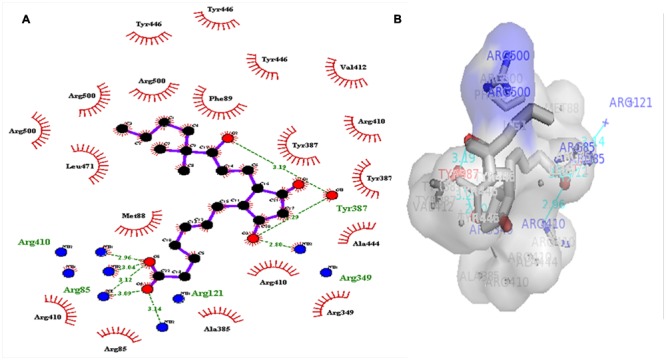
**2D (A)**, and 3D **(B)** representation of docked structure of COI1 generated by Ligplot and PyMol; The 2D representation of 3D structure depicted H-bond interaction of ZINC43772052 with COI1 amino acid residues, ARG85, ARG121, ARG349, TYR387, and ARG410 (green line) whereas the amino acid residues ARG85, MET88, PHE89, ARG349, ALA385, TYR387, ARG410, VAL412, ALA444, TYR446, LEU471, and ARG500 were interacted through hydrophobic bonding (in red color).

**FIGURE 7:**
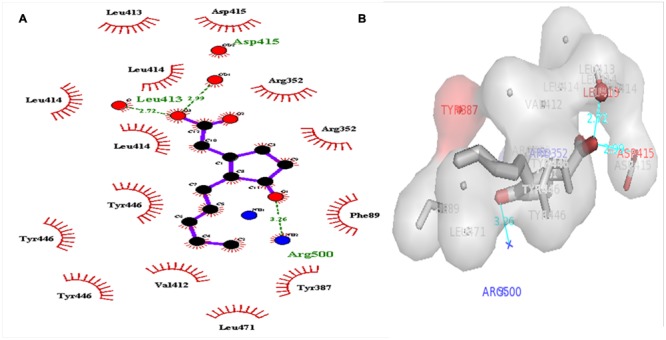
**2D (A)**, and 3D **(B)** representation of docked structure of COI1 generated by Ligplot and PyMol; The 2D representation of 3D structure depicted H-bond interaction of JA with COI1 amino acid residues, ASP415, LEU413, and ARG500 (green line) and some amino acid residues such as PHE89, ARG352, TYR387, VAL412, LEU413, LEU414, ASP415, TYR446, and LEU471 were involved in protein–ligand interaction through hydrophobic bonding (in red color).

A dataset of JA and its structural analogs, which comprises of total 768 small molecule including JA have been used for virtual screening of COI1 (JA receptor) using AutoDock vina (Trott and Olson, 2010). Top two ligand molecules, whose binding energy is higher along with JA, were considered for further analysis (**Figures 5**–**7**). The top scoring compound ZINC27640214 was having binding energy of -7.0 Kcal/mol and the second top compound ZINC43772052 showed binding energy of -6.9 Kcal/mol. In comparison with above identified compound, the naturally occurring JA (**Figure 2B**) showed binding energy of -5.5 Kcal/mol. Details about binding free energy, number of hydrogen bond as well as interacting amino acid of top selected compound along with JA are shown in **Table 4**.

**Table 4:** Summary of results of docking analyses of selected compounds with amino acid residues of COI1 as target involved in protein–ligand interactions through hydrogen bonding with number of H-bond.

S. no.	Ligand (s)/ID	Binding free energy (Kcal/mol)	No. of H bond	Amino acid residues involved in protein–ligand interactions
1	ZINC27640214	–7.0	8	ARG^85^, ARG^121^, ARG^349^, ARG^410^, ARG^500^
2	ZINC43772052	–6.9	8	ARG^85^, ARG^121^, ARG^349^, TYR^387^, ARG^410^
3	Jasmonic acid	–5.5	3	ASP4^15^, LEU^413^, ARG^500^

### Molecular Dynamics Simulation

Diverse crucial biological aspects such as conformational behavior of proteins (Balasco et al., 2015), knowledge about structural insights (Fan et al., 2015) and investigation of novel molecules and its stability with molecular target (Arfeen et al., 2015) can be studied *via* introducing atomic-level perturbations using MD simulations analysis. A molecular dynamics simulation time step comprises of a computationally intensive force calculation for each of the atom in a system, followed by a less expensive integration step which advances the positions and dynamical behavior of the atoms according to classical laws of motion. It allowed us to decoding the atomic-level features of bimolecular processes such as stability analysis of protein and ligands molecules during its interactions linked with activation and deactivation of various molecular pathways associated with diverse biological processes. The stability analysis of protein (COI1) and protein–ligand (COI1-JA, COI1-ZINC43772052, COI1 ZINC27640214) complexes were done by surrounding into a cubic box where a temperature of 300 K was maintained computationally. System was solvated using SPC water model and 3 Na^+^ ions were added into the systems for neutralizations and finally, the production run was executed at 50 ns time scale after system got their equilibrium stage. Various computational analyses were carried out to check the stability of the systems (Van Der Spoel et al., 2005).

#### Root Mean Square Deviation (RMSD) Analysis

**FIGURE 8:**
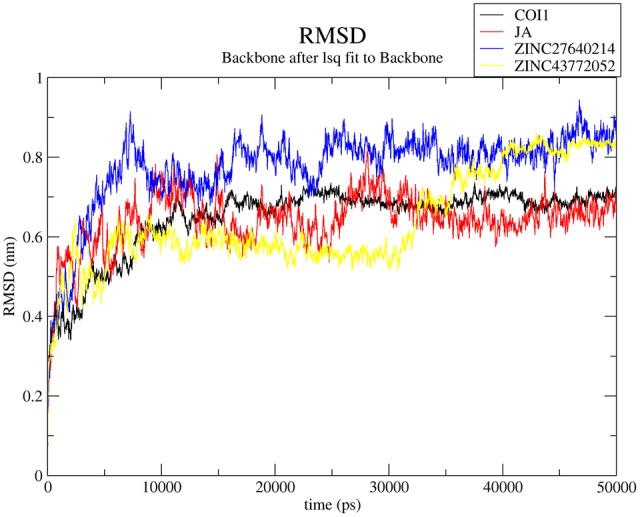
**Time dependent root mean square deviation (RMSD) of C-α backbone of the COI1 and its complexes.** Black, red, blue, and yellow color represents COI1, COI1-JA, COI1-ZINC27640214, and COI1-ZINC43772052, respectively.

RMSD (root mean square deviation) of the complexes with respect to initial unbound structure was plotted in **Figure 8** for predicting the stability of the complexes with respect to initial bound structure. RMSD is used to measure the scalar distance between atoms in a structure. All the systems were well-equilibrated after 35 ns and produced stable trajectory for analysis. The COI1-JA complex showed the average RMSD is 0.63 nm which is lower than apo-form of COI1 because protein showed 0.69 nm. This value justifies the greater stability of COI1-JA complex. RMSD of COI1-ZINC27640214 showed average value of 0.82 nm while COI1-ZINC43772052 showed 0.51 nm till 30 ns but after that it gets instability and RMSD peak showed abrupt pattern of RMSD. But after 40 ns RMSD value was constant and showed 0.83 nm. RMSF, Hydrogen bonds, PCA and binding free energy was calculated for last 10 ns trajectory only.

#### RMSF and Hydrogen Bond Analysis

**FIGURE 9:**
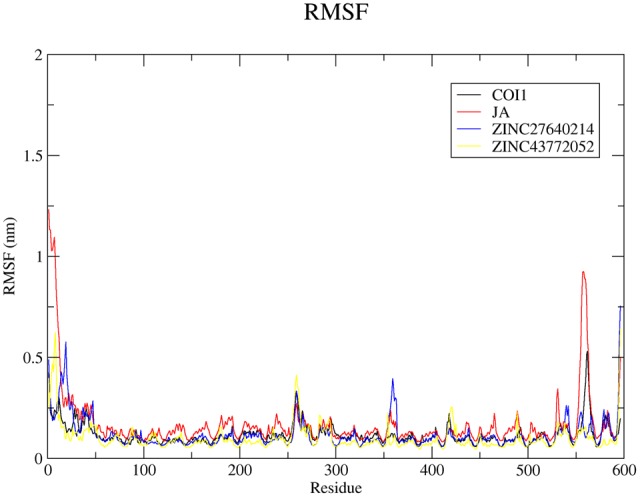
**The root mean square fluctuation (RMSF) for c-α atoms of COI1 and its complexes of last 10 ns trajectory.** Black, red, blue, and yellow color represents COI1, COI1-JA, COI1-ZINC27640214, and COI1-ZINC43772052, respectively.

The root mean square fluctuation (RMSF) analysis discloses the information about movement of atoms in the flexible regions of protein during ligand binding. Overall analysis of RMSF plots for COI1 and JA complex as well as with two best scoring compounds obtained from virtual screening was plotted in **Figure 9**. Average RMSF value of C-α for COI1, COI1-JA, COI1-ZINC27640214, and COI1-ZINC43772052 was 0.11, 0.13, 0.12, and 0.10 nm, respectively.

**FIGURE 10:**
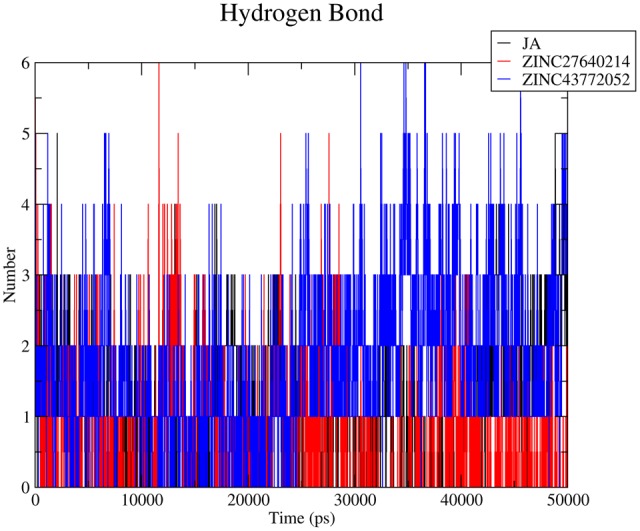
**Number of hydrogen bonds was calculated for evaluation of protein–ligand interaction with respect to time of the COI1-JA, COI1-ZINC27640214, and COI1-ZINC43772052**.

Hydrogen bond plays a vital role during protein–ligand interaction to maintain the stability of the complex. Number of Hydrogen bonds was calculated of whole trajectory which was shown in **Figure 10**. Average number of hydrogen bonds was 3 for COI1-ZINC27640214 and COI1-ZINC43772052. Natural substrate JA showed less number of hydrogen bonds 2 as compare to predicted top two molecule. Interacting residue atoms with ligand was calculated and shown in Supplementary Table S1. After that exploring the key residues which are playing a key prominent role during ligand binding percent occupancy was calculated (Supplementary Table S1).

#### Principal Component Analysis

**FIGURE 11:**
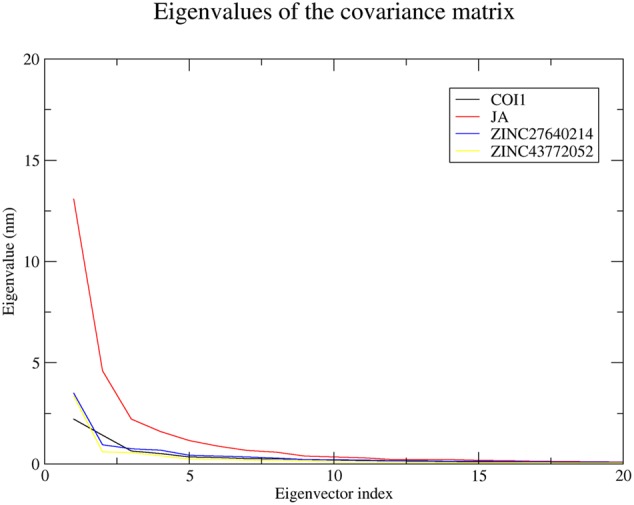
**The eigenvalues plotted of the first 20 eigenvector obtained from the C-α covariance matrix constructed from last 10 ns trajectory**.

Significant concerted motions during ligand binding were calculated for COI1 and its docked complexes using principle component analysis or essential dynamics method. It describes the atomic motions. It is well-known that first few eigenvector captures the essential dynamics of the system. 20 principal components were selected for calculation. From this analysis we found that first four principal component can account for 64.59, 78.75, 69.57, and 73.42% of the motions observed in last 10 ns trajectory for COI1, COI1-JA, COI1-ZINC27640214, and COI1-ZINC43772052, respectively. **Figure 11** showed that each system showed differences in the dynamics behavior. We have found that COI1 showed less correlated motions. But this dynamics is changed during JA binding. JA binding cause the higher motions in the protein. ZINC27640214 and ZINC43772052 showed same type of correlation motion during binding. This result suggests that ZINC27640214 and ZINC43772052 binding is novel as compare to JA.

#### Binding Free Energy Analysis

Binding free energy of protein ligand complex was calculated and shown in **Table 5**. Our result clearly indicates that ZINC27640214 showed greater binding energy (-344.067 Kcal/mol) as compare to known substrate JA (-265.008 Kcal/mol) and ZINC43772052 (-141.503 Kcal/mol). But ZINC43772052 showed less binding energy as compared to JA.

**Table 5:** Van der Waals interaction, electrostatic, polar salvation, SASA, binding energy in kJ/mol for selected docked ligand.

Conformations	Van der Waals/kJ/mol	Electrostatic/kJ/mol	Polar salvation/kJ/mol	SASA/kJ/mol	Binding energy/kJ/mol
ZINC27640214	–180.650 ± 12.761	–437.862 ± 58.439	293.351 ± 76.045	–18.906 ± 1.371	–344.067 ± 56.660
ZINC43772052	–157.130 ± 14.502	–384.089 ± 57.352	418.686 ± 107.116	–18.970 ± 1.273	–141.503 ± 63.938
JA	–46.928 ± 13.934	–455.930 ± 57.423	245.737 ± 100.487	–7.887 ± 1.801	–265.008 ± 76.732

### Physicochemical Properties and Drug Likeness Analysis

**FIGURE 12:**
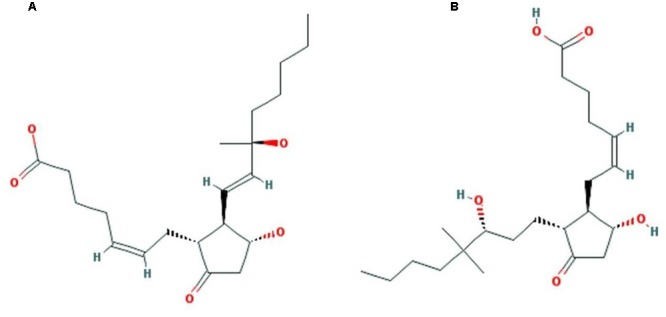
**Structure of top two identified molecules through virtual screening. (A)** ZINC27640214 **(B)** ZINC43772052.

**FIGURE 13:**
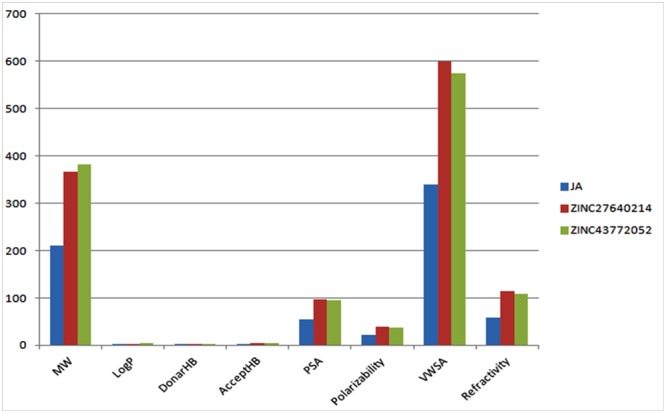
**Values of principal descriptors for JA, ZINC27640214 and ZINC43772052**.

The physicochemical properties of identified molecule along with JA was taken from pubchem and ZINC database as well as predicted by MarvinSketch to evaluate the drug likeness (**Figure 12**). The total eight principal descriptors were included in the study: molecular weight (MW), LogP, H-Bond donor (DonorHB), H-Bond acceptor (AcceptHB), Polar Surface Area 2D (PSA), Polarizability, Van der Waals Surface Area 3D (VWSA), and Refractivity (Pathak et al., 2016) to deciphered the chemical behavior of molecules. The JA and its identified analogs ZINC27640214 and ZINC43772052 has possessed PSA value less than 140 Å, it was predicted that they have good cell membrane permeability. On the other hand, the molar refractivity between 40 and 130 is an indication of better molecules with respect to agrochemical or drug. The top selected compounds ZINC27640214 and ZINC43772052 having molecular weight 365.49 and 381.533, LogP 3.31 and 4.03, H-bond donor 2 and 2, H-bond acceptor 5 and 5, PSA 97.66 and 94.50, Polarizability 39.92 and 37.78, VWSA 598.75 and 574.32, and Refractivity 114.91 and 108.28, respectively (**Figure 13** and **Table 6**).

**Table 6:** Physiochemical properties [chemical formula, molecular weight, LogP, H-bond donor and acceptors, polar surface area in (2D), polarizability, Van der Waals surface area in (3D and refractivity) of Jasmonic acid] and its selected analogs.

Properties	JA	ZINC27640214	ZINC43772052
Molecular weight (g/mol)	210.273	365.49	381.533
LogP	2.41	3.31	4.03
H-bond donor	1	2	2
H-bond acceptor	3	5	5
Polar surface area (2D) (Å)	54.37	97.66	94.5
Polarizability	22.46	39.92	37.78
Van der Waals surface area (3D) (Å^2^)	340.04	598.75	574.32
Refractivity	58.56	114.91	108.28

## Discussion

In recent years, increasing demand of oilseeds has drawn more attention to produce sufficient amount of oils for rapidly growing world population. Besides, the oilseed production has been a backbone of several agricultural economies from the past time and played a significant role in agricultural industries and trade all over the world (Gupta, 2015). Oil from the seeds of crop plants belonging to the family Cruciferae, genus *Brassica*, have been used by man from 1000s of the years for various domestic purposes (Prakash and Hinata, 1980). During last 30 years, *Brassica* oilseed crops have received international importance due to its oil ingredients and nutritional properties (Lamb, 1989). Out of the 37 species in the *Brassica* genus, the four species namely, *B. rapa* L., *B. juncea* (L.) Czernj and Cosson, *B. napus* L., and *B. carinata* A. are widely cultivated as a source of oilseed and vegetable (Sovero, 1993; Raymer, 2002; Rakow, 2004; Gupta, 2015), but these crops are susceptible to a number of diseases caused by several pathogens. Among various diseases, *Alternaria* blight is the most destructive with no known source of resistance available so far. The chemical control of disease is biohazardous and costly. It is believed that the disease can be controlled successfully by developing some novel fungicides in the form of defense inducers which can mimick the action of various plant hormones in triggering the plant’s own immune response. Traditionally, necrotrophic fungal pathogens have been shown to be the primary activators of JA dependent defenses through activation of the receptor, COI1 (Antico et al., 2012). Recent studies demonstrated that the *Fusarium oxysporum* hijacks COI1-mediated JA signaling to promote disease development in *Arabidopsis* (Thatcher et al., 2009). With the availability of complete sequence information of COI1 for the host *B. rapa* and chemical compounds similar to JA, it has become possible to identify novel defense inducer molecules that can mimick JA signaling pathway in more efficient manner that naturally occurring JA through interaction with COI1 during pathogenesis process. Application of such defense inducers will help in development of *de novo* resistance in *Brassica* species against *Alternaria* blight disease for increasing productivity and sustainability of *Brassica* crops. In the present study, computational approaches have led to identification of defense inducer molecules for triggering JA mediated immunity through interaction with COI1 against the infection of *Alternaria* species on *Brassica* crops.

The sequence analysis of COI1 at nucleotide and protein level revealed its nucleotide density, A-T, C-G rich region and physicochemical properties included molecular weight, amino acid composition, theoretical pI, aliphatic index, instability index, and grand average of hydropathicity (GRAVY) were found in appropriate range for influencing the stability of protein (Khurana et al., 1995; Gasteiger et al., 2005). These computational analyses along with secondary structure and BLASTp based structural annotation of COI1 protein sequence at RCSB Protein Data Bank led to the development of comparative model through homology modeling algorithms for novel insights to the identification of mimicking molecules (Bernstein et al., 1978; Altschul et al., 1990; Berman et al., 2003; Pathak et al., 2016). Comparative modeling of the protein is considered as one of the most precise methods for prediction of 3D structure, yielding suitable models for a broad spectrum of applications in area of natural sciences (Bodade et al., 2010; Pathak et al., 2016). It is usually an algorithm of choice when a clear association of homology between the target protein sequence and minimum one known structure is found in RCSB PDB database^5^. This approach would give us a reasonable results based on the assumption that the three dimensional structure of two proteins will be similar, if their amino acid sequences are related. A higher sequence identity assures a more reliable alignment between the sequence of target and the template structure. Therefore, a 3D structural model of COI1 was developed and analyzed through several computational tools to accesses the stereochemical quality of our predicted protein model to understand its reliability. Generating 3D structures of protein from sequence information, in the absence of experimentally determined structures in protein data bank through computational approaches is a priority for the scientific community based on structural biology research since several decades (McGuffin et al., 1999; Hekkelman et al., 2010; Bagaria et al., 2012). Accurate protein models are crucial for virtual screening or identification of agrochemicals, because the accuracy of protein model determines the range of its potential applications. If we are unsure about the quality of protein model obtained from experimental techniques such as X-ray crystallography, NMR, Spectroscopy or predicted from computational methods such as homology modeling, threading or Ab initio are meaningless to used for identification of novel molecules through virtual screening or molecular docking (Snyder et al., 2005; Tramontano and Lesk, 2006). So, the structure of COI1 model was subjected to Structural Analysis and Verification Server (SAVES)^11^ for quality checking, subsequently structural refinement was done to improve the quality of model through energy minimization. The main objective of energy minimization is to find out the lowest energy of molecules with its stable conformation, then further SAVES analysis was performed to confirmed the quality of model followed by ProSA, and superimposition analysis with experimentally determined template structure as well as atoms RMSD assessment to obtained a high quality structural model for virtual screening (Vidya et al., 2008).

Virtual screening can provide precious support in discovery of novel molecules; some software’s have been developed for this purpose (Schneider, 2010). In several drug discovery projects, the virtual screening technology has been the key contributor to find out new ligands on the basis of biological structures and their binding site residues (Shoichet, 2004; Schneider, 2010). The predicted binding site area of COI1 was targeted in present study to evaluate the binding affinity of JA and its structural analogs through virtual screening. Total of 768 molecules was screened along with JA to investigate the new molecules on the basis of their binding energy with COI1. The top identified molecule ZINC27640214 formed eight hydrogen bonds with amino acid residues ARG85, ARG121, ARG349, ARG410, and ARG500 whereas the amino acid residues such as ARG85, ALA86, MET88, PHE89, LEU91, ARG349, TYR387, ARG410, VAL412, ALA444, TYR446, LEU471, GLU498, ARG500, and TRP523 were also interacted through hydrophobic bonding (**Figure 5**). The second top identified molecule ZINC43772052 also form eight hydrogen bond with amino acid residues ARG85, ARG121, ARG349, TYR387, and ARG410 whereas the amino acid residues ARG85, MET88, PHE89, ARG349, ALA385, TYR387, ARG410, VAL412, ALA444, TYR446, LEU471, and ARG500 were also interacted through hydrophobic bonding (**Figure 6**). While, in comparison with these molecules, naturally occurring JA formed only three hydrogen bond with amino acid residues ASP415, LEU413, and ARG500 and some amino acid residues such as PHE89, ARG352, TYR387, VAL412, LEU413, LEU414, ASP415, TYR446, and LEU471 were also involved in protein–ligand interaction through hydrophobic bonding (**Figure 7**). To understand the dynamic behavior of COI1 and top two identified molecules docked with COI1, protein–ligand complex were subjected to Molecular dynamics simulation studies for stability analysis. It accelerate the prediction with lowest error and data loss as well as offers the fluctuations in the relative positions of the atoms present in a proteins and protein–ligand complex (Karplus and Petsko, 1990; Karplus and McCammon, 2002). During simulation a cubic box *size is 11.48 nm × 11.48 nm × 11.48 nm* and temperature of 300 K was maintained computationally. It has 47742 water molecules with protein. COI1 protein has many acidic amino acids so for that reasons our aim is to neutralize the system. Our all systems showed -3 negative charge so for neutralizing the system we added 3 Na^+^ ions only and subsequently the production run was executed for 50 ns time scale to understands the dynamic behaviors of COI1, COI1-JA and COI1- ZINC27640214 as well as COI1-ZINC43772052 complex at atomic level through RMSD, RMSF, PCA, and Hydrogen bond as well as binding free energy analysis. RMSD value reveals that COI1-JA complex was more stable as compare to predicted ligand complex, because it is a natural substrate of protein. In the both predicted ligands, the ZINC27640214 is found better as compare to ZINC43772052 (**Figure 8**). The lowest value of RMSF for COI1-ZINC43772052 reveals that ligand did not cause the more fluctuation during binding. The ZINC43772052 showed lower fluctuation as compare to COI1 and COI1-ZINC27640214. From average RMSF value, we concluded that our both predicted defense inducer molecule was well fit in to the binding cavity and do not cause the much fluctuation in the protein cavity (**Figure 9**). Hydrogen bond calculation analysis showed that the amino acid residues TYR446, LYS496, and ARG85 plays key role during JA stabilization, whereas, Ala86 provides the stability during COI1-ZINC27640214 binding and ALA87, TYR387, GLU351 plays key role during the stabilization of COI1-ZINC43772052 complex (**Figure 10**). The results of PCA suggest that the binding of ZINC27640214 and ZINC43772052 is novel as compare to JA (**Figure 11**). From the results of binding free energy analysis obtained from molecular dynamics simulation, we concluded that the ZINC27640214 may be able to activate the pathway of JA to develop resistance in *Brassica* during the infection of *Alternaria* species.

As per Lipinski’s rule of five a drug will illustrate good ADME (absorption, distribution, metabolism, and excretion) properties if it’s logP value is less than 5, Hydrogen bond donor should be less than 5, Hydrogen bond acceptor should be less than 10 and Molecular weight should be less than 500 (Lipinski et al., 2001). A molecule has less than 140 Å of PSA showed good cell membrane permeability. The physicochemical properties and drug likeness of JA and its identified analogs have shown physicochemical properties according to the Lipinski. Therefore, which may be able to easily enter in plant cell through the stomata and might be play crucial role for triggering JA mediated immunity during pathogenesis (**Figures 12, 13**) (Walter, 2002; Pathak et al., 2013a,b, 2016; Kumar et al., 2015).

In 1960s more than 1 kg of agrochemical was generally applied per ha due to lack of knowledge about the potential molecular target, today the use rates can be considerably reduced as 10 g/ha, it is only 1% of that previously required because of advances in structural biology and use of bioinformatics tools for identification of novel, efficient and potent molecules (Schirmer et al., 2012; Lamberth et al., 2013; Pathak et al., 2016). The results of present study clearly revealed that the JA analog ZINC27640214, could act as a lead molecule as defense inducer for the prevention and management of *Alternaria* blight disease of *Brassica*. ZINC27640214 is showed greatest binding affinity along with hydrogen bond interaction as compare to other compounds selected in this study, it could cross cell membranes due to ideal logP value and low molecular weight as well as its hydrophobic nature, and are able to triggering JA mediated immunity in *Brassica* by interaction with COI1 for production of antimicrobial compounds to develop a resistant systems that controlling crop systems and maintaining its integrity during *Brassica–Alternaria* Interaction to destroy effect of *Alternaria* toxins. It might be also useful for protection of other crops against the infection of plant pathogens.

## Conclusion

The present computational study provides an insight about the interactions between JA and its analogs with COI1 of *B. rapa.* We have concluded that JA showed good stability as compare to its predicted analogs. Whereas predicted analogs were found excellent in RMSF, Number of Hydrogen bonds and Principal component analysis. ZINC27640214 showed greater binding affinity as compare with JA and ZINC43772052. Our finding suggests that the ZINC27640214 is able to work as a mimicking molecule to triggering JA mediated immunity during *Alternaria* infection to prevent and manage *Alternaria* Blight of *Brassica* for securing food and nutritional security of the rapidly growing world population. However, field trial is required to validate its efficacy and potency to provide new molecule for farmers that will directly replace the use of hazardous fungicide, and maintaining human health and soil.

## Author Contributions

AK conceptualized the idea and supervised all the experiments; RKP performed all the experiments, interpreted the results and formulated the manuscript. RS helped in Molecular Dynamics Simulation. MB, RS, DP, GT, and AK critically read the manuscript and provided valuable inputs; all authors read and approved the final manuscript.

## Conflict of Interest Statement

The authors declare that the research was conducted in the absence of any commercial or financial relationships that could be construed as a potential conflict of interest.

## Footnotes

1 http://www.ncbi.nlm.nih.gov/

2 https://in.mathworks.com/products/bioinfo/

3 http://web.expasy.org/cgi-bin/protparam/protparam

4 https://npsa-prabi.ibcp.fr/cgi-bin/npsa_automat.pl?page=/NPSA/npsa_sopma.html

5 http://www.rcsb.org/

6 https://services.mbi.ucla.edu/SAVES/

7 http://spdbv.vital-it.ch/

8 http://zhanglab.ccmb.med.umich.edu/COACH/

9 https://www.ncbi.nlm.nih.gov/pccompound

10 http://zinc.docking.org/

11 http://services.mbi.ucla.edu/SAVES/
