# Supplementary material for: In Silico Identification of Mimicking Molecules as Defense Inducers Triggering Jasmonic Acid Mediated Immunity against Alternaria Blight Disease in Brassica Species
Source: Front Plant Sci. 2017 Apr 25;8:609. doi: 10.3389/fpls.2017.00609 (PMC5403927; doi:10.3389/fpls.2017.00609)
Supplement: Supplementary file 1 [file Table_1.DOCX]

**Supplementary Table S1:**

The occupancy of H-bonds formed between β-tubulin of COI1 and the ligand molecule, during the entire simulation time period (50 ns); Bold indicates the more occupancy of H-bond formation.

| **S.N.** | **Amino acid residue Atom** | **JA**  **Atom** | **% Occupancy** | **Amino acid residue Atom** | **ZINC27640214 Atom** | **% Occupancy** | **Amino acid residue Atom** | **ZINC43772052**  **Atom** | **% Occupancy** |
| --- | --- | --- | --- | --- | --- | --- | --- | --- | --- |
|  | ASP415-Main-N | OAJ | 0.04% | ARG85-Side-NH2 | OAY | 0.08% | ARG85-Side-NH2 | OAZ | 0.04% |
|  | ARG500-Side-NH1 | OAA | 0.08% | ARG85-Side-NH2 | OAX | 0.12% | **ARG410-Side-NH1** | OAF | 2.12% |
|  | **TYR446-Side-OH** | OAI | 1.84% | ARG85-Side-NE | OAX | 0.08% | ARG85-Side-NH1 | OAZ | 0.04% |
|  | ARG85-Side-NH2 | OAA | 0.04% | ARG85-Side-NE | OAY | 0.12% | ARG85-Side-NH1 | OAY | 0.04% |
|  | **TYR446-Side-OH** | OAJ | 3.16% | LYS81-Side-NZ | OAY | 0.04% | ARG85-Side-NE | OAY | 0.08% |
|  | GLN525-Side-NE2 | OAI | 0.04% | ARG410-Side-NH1 | OAE | 0.28% | ARG410-Side-NH1 | OAZ | 0.08% |
|  | **TYR387-Side-OH** | OAA | 1.88% | ARG410-Side-NE | OAE | 0.76% | ARG410-Side-NH1 | OAY | 0.08% |
|  | ARG352-Side-NH1 | OAA | 0.04% | TYR383-Side-OH | OAX | 0.48% | **LYS496-Side-NZ** | OAY | 1.20% |
|  | ARG85-Side-NH2 | OAJ | 0.12% | TYR383-Side-OH | OAY | 0.24% | **LYS496-Side-NZ** | OAZ | 1.08% |
|  | ARG500-Side-NH1 | OAJ | 0.04% | ARG410-Side-NH2 | OAE | 0.16% | LYS81-Side-NZ | OAZ | 0.88% |
|  | ARG410-Side-NH1 | OAA | 0.24% | ARG442-Side-NH1 | OAE | 0.08% | **LYS81-Side-NZ** | OAY | 1.80% |
|  | ARG410-Side-NH2 | OAA | 0.04% | LYS496-Side-NZ | OAX | 0.40% | ALA87-Main-N | OAQ | 0.28% |
|  | ARG85-Side-NE | OAI | 0.28% | ARG120-Side-NH2 | OAY | 0.24% | **ALA87-Main-N** | OAF | 7.71% |
|  | ARG85-Side-NE | OAJ | 0.16% | **ALA86-Main-N** | OAX | 1.56% | ARG120-Side-NH1 | OAY | 0.04% |
|  | ARG121-Side-NH2 | OAA | 0.36% | ARG120-Side-NH2 | OAX | 0.12% | ARG120-Side-NH2 | OAZ | 0.16% |
|  | ARG410-Side-NH1 | OAI | 0.04% | **ALA86-Main-N** | OAY | 4.88% | ARG120-Side-NH1 | OAZ | 0.12% |
|  | ARG410-Side-NH2 | OAI | 0.04% | ARG85-Side-NH1 | OAY | 0.04% | **TYR387-Side-OH** | OAF | 38.61% |
|  | ARG347-Side-NH2 | OAA | 0.12% | ARG120-Side-NH1 | OAY | 0.04% | ARG120-Side-NE | OAY | 0.16% |
|  | ARG347-Side-NH1 | OAA | 0.08% | ARG85-Side-NH1 | OAX | 0.08% | ARG120-Side-NE | OAZ | 0.04% |
|  | ARG347-Side-NE | OAA | 0.32% | ARG500-Side-NH1 | OAP | 0.24% | **GLU351-Side-OE** | OBA | 20.04% |
|  | ARG410-Side-NH2 | OAJ | 0.04% | PHE89-Main-N | OAE | 0.12% | ARG410-Side-NH2 | OAY | 0.08% |
|  | TYR383-Side-OH | OAI | 0.04% | LEU472-Main-O | OAP | 0.04% | ARG85-Side-NH1 | OBA | 0.04% |
|  | ARG407-Side-NH1 | OAA | 0.08% | ARG85-Side-NH1 | OAE | 0.24% | **ARG410-Side-NH1** | OBA | 0.68% |
|  | ARG85-Side-NH1 | OAJ | 0.04% | ARG500-Side-NH1 | OAZ | 0.04% | ARG410-Side-NH2 | OAZ | 0.04% |
|  | ARG120-Side-NH2 | OAA | 0.04% | TRP469-Side-NE1 | OAX | 0.80% | LYS496-Side-NZ | CAX | 0.08% |
|  | TRP469-Side-NE1 | OAJ | 0.16% | TRP523-CZ3 | OAZ | 0.08% | ARG85-Main-O | OAQ | 0.08% |
|  | TRP469-Side-NE1 | OAI | 0.12% | LYS496-Side-NZ | OAY | 0.28% | ARG410-Side-NH1 | OBA | 0.04% |
|  | **GLN495-Side-NE2** | OAA | 2.76% | ALA87-Main-N | OAY | 0.12% |  |  |  |
|  | LYS81-Side-NZ | OAJ | 0.76% | ARG85-Side-NE | OAE | 0.04% |  |  |  |
|  | ARG520-Side-NH1 | OAA | 0.16% | ARG442-Side-NH1 | OAX | 0.08% |  |  |  |
|  | **LYS496-Side-NZ** | OAA | 7.35% | TRP469-Side-NE1 | OAY | 0.48% |  |  |  |
|  | **TRP469-Side-NE1** | OAA | 2.60% | GLN495-Side-NE2 | OAX | 0.08% |  |  |  |
|  | LYS496-Main-N | OAA | 0.04% | GLN495-Side-NE2 | OAY | 0.08% |  |  |  |
|  | LYS496-Side-NZ | OAJ | 0.12% | ARG442-Side-NH2 | OAX | 0.08% |  |  |  |
|  | **ARG85-Main-N** | OAI | 4.04% | ARG85-Side-NH1 | OAZ | 0.04% |  |  |  |
|  | **ARG85-Main-N** | OAJ | 2.56% | ARG410-Side-NH1 | OAY | 0.04% |  |  |  |
|  | **ALA86-Main-N** | OAJ | 2.16% | ALA87-Main-N | OAE | 0.04% |  |  |  |
|  | ARG120-Side-NH1 | OAA | 0.04% | PHE89-Main-O | OAZ | 0.20% |  |  |  |
|  | **LYS79-Side-NZ** | OAA | 1.64% | ARG442-Side-NH1 | OAY | 0.04% |  |  |  |
|  | ARG120-Side-NE | OAA | 0.24% | ARG410-Side-NE | OAX | 0.04% |  |  |  |
|  | GLN56-Side-NE2 | OAJ | 0.20% | ARG85-NH1 | OAZ | 0.04% |  |  |  |
|  | GLN56-Side-NE2 | OAI | 0.08% |  |  |  |  |  |  |
|  | **GLN56-Side-NE2** | OAA | 1.20% |  |  |  |  |  |  |
|  | LYS81-Side-NZ | OAI | 0.16% |  |  |  |  |  |  |
|  | **LYS81-Side-NZ** | OAA | 1.92% |  |  |  |  |  |  |
|  | ARG520-Side-NE | OAI | 0.04% |  |  |  |  |  |  |
|  | LYS79-Side-NZ | OAI | 0.04% |  |  |  |  |  |  |
|  | ARG442-Side-NH1 | OAA | 0.04% |  |  |  |  |  |  |
|  | ALA86-Main-N | OAI | 0.84% |  |  |  |  |  |  |
|  | ARG85-Side-NH1 | OAA | 0.04% |  |  |  |  |  |  |
|  | TYR521-Side-OH | OAJ | 0.16% |  |  |  |  |  |  |
